# Supplementary material for: A malware classification method based on directed API call relationships
Source: PLoS One. 2025 Mar 17;20(3):e0299706. doi: 10.1371/journal.pone.0299706 (PMC11913307; doi:10.1371/journal.pone.0299706)
Supplement: Attachment — Submitted filename: review_PONE-D-23-26475.docx [file pone.0299706.s001.docx]

This research is about “Robust Malware Detection through API-Directed Graph Embeddings”. Title must contain the technique you proposed in the article. And title name is about detection and in the contribution second point classification is mentioned, clear whether this point is detection, classification, or both. However, there's a need for further improvements in terms of clarity, figures, experimental details, and result analysis.

The major concerns in the article are as follows:

Abstract and Introduction:

1. Make the abstract more explicit about suggested improvements or research contributions.

2. Effectively establish the specific research gap or problem.

3. Provide a thorough overview of existing literature.

4. Explicitly state research objectives or hypotheses and the dataset used is not mentioned in the abstract.

5. Organize the introduction more effectively.

6. The contribution of the work is presented ambiguously try to use proper wordings and improve your English to clear your points.

7. what is the meaning of the last paragraph of the introduction part.

Methods:

8. Include more detail regarding model architecture, components, mathematical formulations, and no figure mentioned in the methods.

9. Clearly explain the underlying assumptions of the generalized metric learning model and how they align with the research problem.

10. Justify why the chosen generalized metric learning model was selected over alternatives.

11. Provide specific details about data preprocessing steps.

12. Include the missing figure mentioned in the datasets section.

13. Offer crucial details about dataset sources, characteristics, and relevance to the research question.

14. Specify the chosen evaluation metrics.

15. Provide information about hyperparameter settings, optimization algorithms, and convergence criteria.

Results and Discussion:

14. Clearly state the specific objectives of classification tasks and describe the evaluation protocol.

15. Provide context for what "performed better" means and discuss why (FSADGCN) outperformed other models.

16. The Results section has no figures.

Conclusion and Future Directions:

20. Include specific metrics or quantitative assessments of model performance.

21. Address the feature extraction GCN used in the study and through which CNN model graph classification you have done.

22. Provide a detailed comparison of the proposed approach against existing methods.

23. Discuss potential future research directions and areas for improvement.

24. Conduct an in-depth analysis of the stability of adaptation results.

25. Include qualitative analysis, such as visualizations or examples, to enhance understanding of the model's behavior.
