# Supplementary material for: A malware classification method based on directed API call relationships
Source: PLoS One. 2025 Mar 17;20(3):e0299706. doi: 10.1371/journal.pone.0299706 (PMC11913307; doi:10.1371/journal.pone.0299706)
Supplement: Attachment — Submitted filename: Response to Reviewers.docx [file pone.0299706.s002.docx]

**Dear Reviewers,**

Thank you for your thorough review and valuable feedback on our manuscript "Robust Malware Detection through API-Directed Graph Embeddings." Now, we are changing the title to "Malware classification through API Calls multi-dimensional feature extrate based on SMOTE and Directed Graph convolution network and CNN." We have carefully considered your comments and made the following revisions to our manuscript:

**Reviewer#1 Comment 1:** Title represents the malware detection mechanism. However, the proposed work represents the malware classification. This needs to be addressed first.

**Response:**

Thank you for pointing this out. We have revised the title to more accurately reflect the content of our manuscript. The new title, "Malware classification through API Calls multi-dimensional feature extrate based on SMOTE and Directed Graph convolution network and CNN", aligns with our focus on malware classification rather than detection. This change ensures consistency between the title and the core content of our study.

**Reviewer#1 Comment 2:** The rationale of the proposed work may be elaborated?

**Response:**

We appreciate your suggestion for further elaboration on the rationale of our work. We have expanded the Introduction section to provide a detailed explanation of why our approach was chosen and its significance in the context of current challenges in malware classification. This elaboration includes a discussion on the limitations of existing methods and how our approach addresses these shortcomings. These details can be found in the Introduction section, particularly on pages 1-3.

**Reviewer#1 Comment 3:** Figure are not present while their references are used? please make the Figure for the Framework at least. You can refer to the following paper for the framework diagram

•Khan, S.H., Alahmadi, T.J., Ullah, W., Iqbal, J., Rahim, A., Alkahtani, H.K., Alghamdi, W. and Almagrabi, A.O., 2023. A new deep boosted CNN and ensemble learning based IoT malware detection. Computers & Security, 133, p.103385.

• Asam, M., Khan, S.H., Akbar, A., Bibi, S., Jamal, T., Khan, A., Ghafoor, U. and Bhutta, M.R., 2022. IoT malware detection architecture using a novel channel boosted and squeezed CNN. Scientific Reports, 12(1), p.15498.

**Response:**

Our apologies for the oversight. We have now included figures to visually represent the framework and key concepts of our study. Specifically, a comprehensive framework diagram has been added, inspired by the suggested papers, to illustrate our model architecture and workflow. This figure can be found on page 7.

**Reviewer#1 Comment 4:** Line # 74-80, # 202-207 and similar paragraphs may be explained.

**Response:**

We appreciate your valuable comment and thank you for bringing attention to the confusion matrix. The confusion matrix is part of Figure 9(e). In the revised version, however, we have removed Figure 9(e). This decision was made because we believe that the tables in Table 2 and Table 3 adequately showcase the capabilities of our model, and thus, repetition was deemed unnecessary. We understand the importance of clarity in presenting results, and we will ensure that this information is explicitly conveyed in the revised manuscript. Your feedback is instrumental in improving the overall quality of our work, and we are committed to addressing this concern in the upcoming revision.

**Reviewer#1 Comment 5:** Dataset is highly imbalanced so do you think that measuring the performance as accuracy is better measure?

**Response:**

You raise a crucial point regarding the imbalanced nature of our dataset. To address this, we have employed the Synthetic Minority Oversampling Technique (SMOTE) for resampling, which enhances the representation of minority samples, thus balancing the dataset. This adjustment makes accuracy a more reliable measure. However, we also acknowledge the importance of using a comprehensive set of metrics for imbalanced datasets. Therefore, in addition to accuracy, we have included precision, recall, and F1-score in our evaluation. These metrics provide a holistic view of our model's performance, particularly in the context of the balanced dataset achieved through SMOTE. Details of the SMOTE implementation and its impact on our evaluation metrics can be found in the Resampling of imbalanced samples and Evaluation Metrics subsections on pages 10 and 24, respectively.

**Reviewer#1 Comment6:** Data split for the experiment is not present. Please mention.?

**Response:**

We apologize for this omission and have now included details of the data split used in our experiments. The datasets were split into training, validation, and testing sets in a ratio of 70:15:15. This information is added to the Experimental results subsection on page 20.

**Reviewer#1 Comment 7:** Please use the name of algorithm for [18] and [33] in Table -4.

**Response:**

We have corrected Table 4 to include the names of the algorithms referenced in [18] and [33]. This ensures clarity and ease of reference for readers. The updated table can be found on page 23.

**Reviewer#1 Comment 8:** Confusion matrix referred at Line # 637 is not found. please insert it.

**Response:**

We appreciate your valuable comment and thank you for bringing attention to the confusion matrix. The confusion matrix is part of Figure 9(e). We understand the importance of clarity in presenting results, and we will ensure that this information is explicitly conveyed in the revised manuscript. Your feedback is instrumental in improving the overall quality of our work, and we are committed to addressing this concern in the upcoming revision.

**Reviewer#1 Comment 9:** The performance of “GraphSAGE” is 0.993 which is very close to your proposed methods (0.996). It would be better to perform your experiment with more stringent dataset to prove your stance of Robustness.

**Response:**

Thank you for your insightful observation. Indeed, the initial performance of our method was closely comparable to that of GraphSAGE. However, with the implementation of the Synthetic Minority Oversampling Technique (SMOTE), we achieved significant improvements in all key metrics – accuracy, precision, recall, and F1-score – elevating them to 0.999. This improvement underscores the robustness of our model, especially in handling imbalanced data sets. Moreover, to further validate the robustness and generalizability of our approach, we included an additional dataset, referred to as Dataset 2, in our experiments. This dataset, characterized by its complexity and challenging nature, provided a more stringent testing ground for our method. The results on Dataset 2 corroborate the effectiveness of our approach, demonstrating its adaptability and robustness across diverse and complex malware scenarios. The detailed results and analysis of experiments on Dataset 2 are presented in the Results section on pages 21-24.

**Reviewer#2 Comment 1:**

"Make the abstract more explicit about suggested improvements or research contributions."

**Response:**

Thank you for this suggestion. We have revised the abstract to more explicitly highlight the key contributions of our research. The revised abstract now clearly outlines the integration of the Synthetic Minority Oversampling Technique (SMOTE), Directed-graph Convolutional Network (DGCN), and Convolutional Neural Network (CNN) for enhanced malware classification. It also emphasizes the significant improvements in accuracy and convergence speed achieved by our model. These changes can be found in the Abstract section on pages 1.

**Reviewer#2 Comment 2:**

"Effectively establish the specific research gap or problem."

**Response:**

We appreciate your feedback on this aspect. In the revised introduction, we have expanded our discussion on the limitations of current malware detection techniques, specifically highlighting the research gap in capturing the complexity of malware API call sequences. The revised content providing a detailed elucidation of this research gap is located in the Introduction section on pages 1-3.

**Reviewer#2 Comment 3:**

"Provide a thorough overview of existing literature."

**Response:**

In response to your comment, we have enriched our literature review to provide a comprehensive overview of existing malware detection or classification methods and their shortcomings. This expanded review now offers a more robust context for our research, setting the stage for the novelty of our proposed approach. These enhancements can be found in the Related work section on pages4-6.

**Reviewer#2 Comment 4:**

"Explicitly state research objectives or hypotheses and the dataset used is not mentioned in the abstract."

**Response:**

Thank you for pointing this out. We have amended the abstract to explicitly state our research objectives and hypotheses, and to mention the datasets used in our study. These adjustments ensure that readers are immediately informed about the empirical basis of our research. These revisions can be found in the Abstract section on page 1.

**Reviewer#2 Comment 5:**

"Organize the introduction more effectively."

**Response:**

We have restructured the Introduction section to enhance its organization and flow. The section now systematically progresses from the context of malware threats, to a discussion on existing methodologies, and finally to an introduction of our proposed approach. This reorganization can be found in the Introduction section on pages 1-3.

**Reviewer#2 Comment 6:**

"The contribution of the work is presented ambiguously; try to use proper wordings and improve your English to clear your points."

**Response:**

We acknowledge the importance of this point and have revised the Contribution section for clarity and precision. The language has been improved to precisely communicate the uniqueness and importance of our research in the cybersecurity field. These changes are present in the Contribution section on page 3.

**Reviewer#2 Comment 7:**

"What is the meaning of the last paragraph of the introduction part?"

**Response:**

Thank you for your thorough review and constructive feedback on our manuscript. We appreciate the time and effort you dedicated to evaluating our work. We have identified an oversight in our submission after carefully considering your comments. Certain sections were inadvertently left in the manuscript, originating from the template we used during the initial preparation. We acknowledge this mistake and sincerely apologize for any confusion it may have caused. We have taken immediate steps to rectify this error. The manuscript has been thoroughly reviewed, and the extraneous sections have been removed to align with your constructive suggestions for a more concise presentation. We value your meticulous attention to detail, and your feedback has been instrumental in refining our work. Again, Thank you for your valuable feedback and for being an integral part of the peer-review process.

**Methods:**

**Reviewer Comment 8:**

"Include more detail regarding model architecture, components, mathematical formulations, and no figure mentioned in the methods."

**Response:**

In response to your feedback, we recognize that our model structure was not described in sufficient detail; therefore, we have redrawn a more detailed model architecture diagram (Fig. 1) and provided a comprehensive description of the model architecture. To address the issues related to model components, mathematical formulations, and figures not mentioned in the methods, we have further elaborated on these aspects in the Materials and Methods section, spanning pages 6 to 17.

**Reviewer#2 Comment 9:**

"Clearly explain the underlying assumptions of the generalized metric learning model and how they align with the research problem."

**Response:**

Thank you for this comment. Regarding the reviewer's reference to a 'generalized metric learning model,' our methodology primarily focuses on the application of Directed Graph Convolutional Networks (DGCN) and Convolutional Neural Networks (CNN) for robust malware detection and classification. These techniques are leveraged to effectively capture the complex relationships and features within API call sequences. We did not explicitly employ a generalized metric learning model as part of our approach. If the reviewer could provide further clarification on this aspect, it would greatly assist us in addressing this query more accurately.

**Reviewer#2 Comment 10:**

"Justify why the chosen generalized metric learning model was selected over alternatives."

**Response:**

As mentioned in our response to Comment 9, our method does not directly employ a generalized metric learning model.

**Reviewer#2 Comment 11:**

"Provide specific details about data preprocessing steps."

**Response:**

Since the dataset we obtained has already undergone sandbox processing of API call sequence data, we can directly use it for experimentation. However, the sample sizes for various categories are not balanced, so we applied sample balancing algorithms to resample the data before feeding it into the model for processing. These details are now thoroughly outlined in the Data Preprocessing subsection of the Methods section on pages 9-10.

**Reviewer#2 Comment 12:**

"Include the missing figure mentioned in the datasets section."

**Response:**

We have included the previously missing figure in the datasets section, providing a visual representation of the dataset structure and composition. This figure can be found on page 19.

**Reviewer#2 Comment 13:**

"Offer crucial details about dataset sources, characteristics, and relevance to the research question."

**Response**:

In response to your feedback, we have enriched the dataset description with detailed information about the sources, characteristics, and why they are particularly relevant and suitable for our research question. This comprehensive description is included in the Datasets subsection on pages 18-19.

**Reviewer#2 Comment 14:**

"Specify the chosen evaluation metrics."

**Response:**

We have specified and provided justifications for the choice of evaluation metrics used in our study. These include accuracy, precision, recall, and F1-score, all of which are relevant and suitable for assessing malware classification models. The Evaluation Metrics subsection on pages 19-20 details these metrics.

**Reviewer#2 Comment 15:**

"Provide information about hyperparameter settings, optimization algorithms, and convergence criteria."

**Response:**

Thank you for highlighting this aspect. In response, we have now included comprehensive details about the hyperparameter settings, optimization algorithms, and convergence criteria used in our study. Specific information about the hyperparameters, such as learning rate, batch size, number of layers, and the architecture of the DGCN and CNN models, has been clearly stated. We have also discussed the optimization algorithm (Adam optimizer) used and the criteria for convergence to ensure a thorough understanding of our model's training and validation processes. These details are thoroughly described in the Model Architecture and Training subsections on page 20 of the revised manuscript.

**Results and Discussion:**

**Reviewer#2 Comment 16:**

"Clearly state the specific objectives of classification tasks and describe the evaluation protocol."

**Response:**

We have explicitly stated the specific objectives of our classification tasks in the revised manuscript. These include accurately classifying different types of malware based on API call sequences using our proposed model. The evaluation protocol, including how the datasets were split into training, validation, and testing sets, and the criteria used for evaluation, is now clearly described in the evaluation metrics and experimental results subsection on pages 20-24.

**Reviewer#2 Comment 17:**

"Provide context for what 'performed better' means and discuss why (FSADGCN) outperformed other models."

**Response:**

In the revised Results and Discussion section, we have provided a detailed context for our claim of 'performed better.' This includes comparisons in terms of accuracy, precision, recall, and F1-score against existing methods. We have also discussed the reasons behind the superior performance of our model, attributing it to its effective handling of the sequential nature of API calls and the integration of multi-dimensional features. These discussions are detailed on pages 20-24.

**Reviewer#2 Comment 18:**

"The Results section has no figures."

**Response:**

We have added several figures in the Results section to visually represent our findings. These figures include comparisons of our model's performance against other methods, loss curves, and ROC curves, providing a visual and intuitive understanding of the model's effectiveness. These figures can be found on pages 20-24.

**Conclusion and Future Directions:**

**Reviewer#2 Comment 19:**

"Include specific metrics or quantitative assessments of model performance."

**Response:**

In the Conclusion section, we have included specific metrics and quantitative assessments of our model's performance, such as accuracy, precision, recall, and F1-score. These metrics are now clearly stated to quantify the effectiveness of our model in malware classification. This information is presented on pages 21-24.

**Reviewer#2 Comment 20:**

"Address the feature extraction GCN used in the study and through which CNN model graph classification you have done."

**Response:**

We have addressed the feature extraction process using GCN in our study in the Methods section. We have elaborated on how the GCN is used to extract features from the API call sequences and how these features are subsequently classified using the CNN model. The specifics of this process are detailed on pages 13-19.

**Reviewer#2 Comment 21:**

"Provide a detailed comparison of the proposed approach against existing methods."

**Response:**

A detailed comparison of our proposed approach against existing methods is now included in the Results and Discussion section. This comparison highlights the advantages and improvements of our model over existing malware classification techniques. These comparisons can be found on pages 21-23.

**Reviewer#2 Comment 22:**

"Discuss potential future research directions and areas for improvement."

**Response:**

In the Conclusion and Future Directions section, we have discussed potential future research directions and areas for improvement in our approach. This includes exploring additional datasets, experimenting with different architectures, and further refining our model to enhance its generalizability and robustness. These discussions are detailed on page 24.

**Reviewer#2 Comment 23:**

"Conduct an in-depth analysis of the stability of adaptation results."

**Response:**

An in-depth analysis of the stability of our model's results, particularly in terms of how well it adapts to different datasets and malware types, is now included in the Discussion section. We have examined the consistency of our model's performance across various conditions to demonstrate its stability. This analysis is presented on pages 20-24.

**Reviewer#2 Comment 24:**

"Include qualitative analysis, such as visualizations or examples, to enhance understanding of the model's behavior."

**Response:**

We have included qualitative analyses in the form of visualizations and examples in the Results section. These visualizations help in better understanding the behavior and decision-making process of our model, providing an intuitive grasp of its functionality. Examples of these visualizations are included on pages 20-24.

We trust that these responses and the associated revisions thoroughly address your concerns and enhance the manuscript's quality. We are grateful for the opportunity to refine our work and eagerly await any further feedback.

Best regards,

[Cuihua Ma,Zhenwan Li,Haixia Long,Anas Bilal,Xiaowen Liu]
