# Supplementary material for: A malware classification method based on directed API call relationships
Source: PLoS One. 2025 Mar 17;20(3):e0299706. doi: 10.1371/journal.pone.0299706 (PMC11913307; doi:10.1371/journal.pone.0299706)
Supplement: Attachment — Submitted filename: Response to Reviewers(1).docx [file pone.0299706.s003.docx]

**Dear Reviewers,**

Thank you for your thorough review and valuable feedback on our manuscript "Malware classification through API Calls multi-dimensional feature extrate based on SMOTE and Directed Graph convolution network and CNN". We have carefully considered your comments and made the following revisions to our manuscript:

**Reviewer#2 Comment 1:**

Improve your result and literature section by incorporating the ideas share in the article shared with you in the last review.

**Response:**

Thank you for pointing this out. We have included the papers mentioned in the previous review within the article, see references [4] and [6].

**Reviewer#2 Comment 2:** For expedite the research activities please share the datasets.

**Response:**

Thank you for your suggestion. Below are the links to the datasets and code shared in our paper:https://kaggle.com/datasets/8abfa8c0975603330354e778767d7f269cc4e3e707b6ad92c5ce22acb04ebf7c

**Reviewer#2 Comment 3:** Explain the idea you have incorporated for plotting the ROC curve as your problem is multi class classification.

**Response:**

Thank you for raising this issue. Upon reviewing relevant literature, we concur with your assertion that ROC curves are more suited to binary classification problems. Consequently, we have redrawn the PR curves to illustrate the multi-class problem, as shown in Figure 10.

**Reviewer#2 Comment 4:** Draw PR curve as the dataset is imbalanced.

**Response:**

Thank you for your suggestion. We have included the PR curves, as shown in Figure 10. Figure 10 demonstrates that our model still exhibits superior detection performance in the malware categories with fewer samples, such as Spyware, Adware, and Dropper, compared to categories with a larger number of samples.

**Reviewer#2 Comment 5:** Draw PCA / TSNE based feature space visualization.

**Response:**

Thank you for your suggestion. We have added t-SNE feature space visualizations representing the feature representations obtained from the model when using 1, 3, 5, and 7-dimensional features, along with an analysis of these. See Figure 11 and page 24 of the paper for further details.

**Reviewer#2Comment6:**The validation and train loss curve does not showing convergence which means that the system is going overfitting, explain it.

**Response:**

Thank you for your question. Upon analysis, we indeed find that the loss curve in Figure 13 of the previous version of our paper did not converge, as you pointed out. This was due to the fact that I only presented results for 20 epochs, which was insufficient. In this revision, I have increased the number of training epochs to 200, as depicted in Figure 13. Here, the loss function curve for the validation set tends to stabilize, indicating that the validation set has reached a state of convergence.

**Reviewer#3 Comment 1:** Enhance your literature sections by mentioning the dataset, novelty, framework and integrating the ideas presented in the article provided during the last review.

• Khan, et.al. "A new deep boosted CNN and ensemble learning based IoT malware detection." Computers & Security 133 (2023): 103385.

• Asam, M., et.al, 2022. IoT malware detection architecture using a novel channel boosted and squeezed CNN. Scientific Reports, 12(1), p.15498.

• Zahoora, U., et.al, 2022. Ransomware detection using deep learning based unsupervised feature extraction and a cost sensitive Pareto Ensemble classifier. Scientific Reports, 12(1), p.15647.

•Asam, et.al, A., 2021. Malware classification using deep boosted learning. arXiv preprint arXiv:2107.04008.

• Iqbal, et.al, 2022. An Energy Efficient Local Popularity Based Cooperative Caching for Mobile Information Centric Networks. Sustainability, 14(20), p.13135.

**Response:**

Thank you for pointing this out. We have incorporated the papers mentioned in the previous review into our article, as referenced in [4-8].

We trust that these responses and the associated revisions thoroughly address your concerns and enhance the manuscript's quality. We are grateful for the opportunity to refine our work and eagerly await any further feedback.

Best regards,

[Cuihua Ma,Zhenwan Li,Haixia Long,Anas Bilal,Xiaowen Liu]
