# Supplementary material for: A malware classification method based on directed API call relationships
Source: PLoS One. 2025 Mar 17;20(3):e0299706. doi: 10.1371/journal.pone.0299706 (PMC11913307; doi:10.1371/journal.pone.0299706)
Supplement: Attachment — Submitted filename: Response_to_Reviewers_auresp_3.docx [file pone.0299706.s004.docx]

**Dear Reviewers,**

Thank you for your thorough review and valuable feedback on our manuscript "Malware classification through API Calls multi-dimensional feature extrate based on SMOTE and Directed Graph convolution network and CNN". We have carefully considered your comments and made the following revisions to our manuscript:

**Reviewer#4 Comment 1:**

Please give out the core algorithm pseudo-code for proposed model.

**Response:**

Thank you for pointing this out. We have added the pseudocode of the entire algorithm at the end of the Materials and Methods section in the paper.

**Reviewer#4 Comment 2:** The complexity of the algorithm is also discussed.

**Response:**

Thank you for your suggestion. We added a short section discussing the time complexity of the algorithm at the end of the Experimental Results section.

**Reviewer#4 Comment 3:**

Some related references are missing as follows:

Naeem H, Cheng X, Ullah F, et al. A deep convolutional neural network stacked ensemble for malware threat classification in internet of things[J]. Journal of Circuits, Systems and Computers, 2022, 31(17): 2250302.

Naeem H, Dong S, Falana O J, et al. Development of a deep stacked ensemble with process based volatile memory forensics for platform independent malware detection and classification[J]. Expert Systems with Applications, 2023, 223: 119952.

Shu L, Dong S, Su H, et al. Android malware detection methods based on convolutional neural network: A survey[J]. IEEE Transactions on Emerging Topics in Computational Intelligence, 2023.

Dong S, Shu L, Nie S. Android Malware Detection Method Based on CNN and DNN Bybrid Mechanism[J]. IEEE Transactions on Industrial Informatics, 2024.

**Response:**

Thank you for pointing this out. We have added citations for these articles in the Introduction section of the paper, as referenced in [2][8-10].

**Reviewer#5 Comment 1:** I don’t understand that why GCN is still necessary, there are many powerful architectures, such as transformer and mamba, etc. Why you combine GCN and CNN?

**Response:**

Thank you for pointing this out. This approach was chosen because, based on the issues this paper seeks to address, we identified that some current methods for malware detection using API sequences tend to overlook the structural and directional information within the sequences. To address this limitation, we selected GCN due to its effectiveness in handling tasks involving graph-structured data. Given that the graph representation features we obtained are in the form of a two-dimensional matrix, converting this matrix into a grayscale image allows us to leverage CNN, which is highly proficient in image processing, for classification. Consequently, we integrated GCN and CNN in our approach. We appreciate your suggestion and, in future work, we will explore research within the frameworks of Transformer and Mamba.

**Reviewer#5 Comment 2:** The dimension of the dataset should be mentioned, if the dimension is not great, why not directly use MLP.

**Response:**

Thank you for pointing this out. Since an API sequence is equivalent to a piece of malware, the latent representation obtained through GCN is a two-dimensional feature. This feature is then converted into a grayscale image, and CNN, which excels in image classification, is used for the classification task. Essentially, we have constructed the API sequence as a graph and then performed graph classification. Although MLP can also handle classification tasks, it may require further processing of the API sequence graph embeddings we obtained, such as applying global average pooling, before classifying the processed data. We believe your suggestion is valuable, and in the next step, we will explore using MLP for classification in our research.

**Reviewer#5 Comment 3:** Please polish the paper to enhance the flow and improve the language.

**Response:**

Thank you for your valuable feedback. We will carefully revise the manuscript to improve its flow and enhance the language. We understand the importance of clear and concise communication in academic writing, and we will make the necessary adjustments to ensure that the paper meets the highest standards of readability and coherence.

**Reviewer#5 Comment 4:** Several key works are not mentioned, even they are highly related to this work. For example, “Hypernetwork-based physics-driven personalized federated learning for CT imaging”, “Physics-Driven Spectrum-Consistent Federated Learning for Palmprint Verification” and “FCSCNN: Feature centralized Siamese CNN-based android malware identification”.

**Response:**

Thank you for pointing this out. We have added citations for these articles in the Introduction section of the paper, as referenced in [12-14].

**Reviewer#5 Comment 5:** Please enhance the image quality.

**Response:**

Thank you for your suggestion. We will improve the image quality in the revised version of the paper to ensure that all visual elements are clear and of high resolution.

**Reviewer#5 Comment 6:** The equation is not well described. For example, in Eq. 1, i is the webpage, this variable should be the subscript. I do believe the input of PR is not only the index, right?

**Response:**

Thank you for pointing this out. In response to your concerns, we have made revisions to the manuscript, such as in Equation 11. To better align with the content of our research, the original variable $i$ has been changed to $v_{i}$, and the meanings of the other parameters have been adjusted accordingly.

**Reviewer#5 Comment 7:** The formula format is not standardized. When the formula variables are not clearly explained, a comma should follow the formula, and a "where" should be placed below it. The variables should then be explained within a fixed frame.

**Response:**

Thank you for your feedback. We have reviewed the formula formatting throughout the manuscript and made revisions according to your suggestions. We hope that the updated format meets the requirements.

**Reviewer#5 Comment 8:** Why do you compare methods that are different in different datasets? Can you reproduce these methods following their papers?

**Response:**

Thank you for pointing this out.In Table 2, we compare our method with MLP, N-gram, LSTM, and SVM on two datasets. These four methods were implemented in our code, and applying them to both datasets was done to enhance the generalization ability of our algorithm. Table 3 presents a comparison with algorithms from other papers. The methods SDGNet, Mal-ASSF, and LGMal all used Dataset 1, so we directly used the data from those papers for comparison. Catak et al. [50] used Dataset 2, so we also used the data from their paper for comparison.

**Reviewer#5 Comment 9:** As you said, SMOTE is designed to alleviate the unbalanced problem. The key ablation study about this is missing, and please show the results w/ and w/o SMOTE following Fig. 11.

**Response:**

Thank you for your feedback. In Table 6 of the Ablation Studies subsection within the Results and Discussion section, we conducted an analysis w/ and w/o SMOTE

**Reviewer#5 Comment 10:** Besides, I think SMOTE must lead to an overfitting problem, please discuss it. I think this problem is very important, unless you can discuss it sufficiently, or this method is not convincing.

**Response:**

Thank you for pointing out the issue. Based on your observation, we found that there was indeed overfitting in the comparison of the loss functions between w/o SMOTE and w/ SMOTE (originally Fig 13). To address this, we increased the regularization strength in the CNN model, and the overfitting issue has now been significantly improved, as shown in Fig 10.

We trust that these responses and the associated revisions thoroughly address your concerns and enhance the manuscript's quality. We are grateful for the opportunity to refine our work and eagerly await any further feedback.

Best regards,

[Cuihua Ma,Zhenwan Li,Haixia Long,Anas Bilal,Xiaowen Liu]
