# Supplementary material for: A malware classification method based on directed API call relationships
Source: PLoS One. 2025 Mar 17;20(3):e0299706. doi: 10.1371/journal.pone.0299706 (PMC11913307; doi:10.1371/journal.pone.0299706)
Supplement: Attachment — Submitted filename: PONE-D-23-26475.docx [file pone.0299706.s005.docx]

**Reviewer for Manuscript Number PONE-D-23-26475
"A Malware Classification Method Based on Directed API Call Relationships**

**Comments:**

Overall paper is well structured. However, address following points for further improvements.

- The abstract doesn’t address how the method handles real-world scenarios like noisy or imbalanced datasets.
- The introduction briefly mentions real-world scenarios but could benefit from elaborating on how the method handles challenges like imbalanced datasets or novel malware does not present in training data.
- Ensure consistent use of terms like "graph convolutional networks (GCN)" and "directed graph convolutional networks (DGCN)" across sections to avoid confusion.
- Some references, such as those for malware statistics, could be better integrated into the discussion to highlight their relevance to the problem statement.
- Ensure all abbreviations like FSADGCN, CNN, and API are defined explicitly when first introduced. For example, "API sequence instructions" can be expanded to "Application Programming Interface (API) sequence instructions."
- Replace the placeholder text "Fig 1 venenatis sed ipsum varius..." with an appropriate description or remove unrelated filler text. Ensure figure references are meaningful and properly aligned with the content.
- Rephrase sentences to improve readability, such as changing "The extracted node feature attributes are then subjected to..." to "Next, the extracted node feature attributes undergo..."
- Review and adjust the formatting of equations to ensure clarity and consistency, such as spacing and alignment in equations (e.g., Equations 1-11). Use inline or displayed math consistently to improve readability.
