# Supplementary material for: A malware classification method based on directed API call relationships
Source: PLoS One. 2025 Mar 17;20(3):e0299706. doi: 10.1371/journal.pone.0299706 (PMC11913307; doi:10.1371/journal.pone.0299706)
Supplement: Attachment — Submitted filename: Response_to_Reviewers_auresp_4.docx [file pone.0299706.s006.docx]

**Dear Reviewers,**

Thank you for your thorough review and valuable feedback on our manuscript "Malware classification through API Calls multi-dimensional feature extrate based on SMOTE and Directed Graph convolution network and CNN". We have carefully considered your comments and made the following revisions to our manuscript:

**Reviewer#6 Comment 1:**The abstract doesn’t address how the method handles real-world scenarios like noisy or imbalanced datasets.

**Response:**

We appreciate your comment about real-world challenges such as noisy or imbalanced datasets. In the revised abstract, we have addressed the handling of imbalanced datasets by incorporating the Synthetic Minority Over-sampling Technique (SMOTE), which ensures that underrepresented classes are sufficiently represented during training.

**Reviewer#6 Comment 2:** The introduction briefly mentions real-world scenarios but could benefit from elaborating on how the method handles challenges like imbalanced datasets or novel malware does not present in training data.

**Response:**

Thank you for your insightful feedback. We appreciate the suggestion to elaborate on how our proposed method handles real-world challenges such as imbalanced datasets and novel malware variants that are not present in the training data. In response to your comment, we have revised the relevant sections of the introduction to provide a clearer explanation of how our method addresses these challenges.

Modifications in the Introduction:

Class Imbalance: We have explicitly addressed the issue of class imbalance, a common challenge in real-world malware classification. In the revised text, we highlight how our approach leverages the Synthetic Minority Over-sampling Technique (SMOTE), a widely used technique for balancing datasets by generating synthetic samples for underrepresented classes. By ensuring a more balanced dataset, SMOTE helps prevent the model from being biased towards the majority class, which in turn improves the model's ability to detect less frequent malware types, including rare or emerging ones.

Novel Malware Detection: We also discuss the challenge of detecting novel malware variants—those that were not observed during training. Our approach's use of a directed graph representation of API call sequences provides a more robust and flexible method compared to traditional static feature-based approaches. This graph-based method better captures the structural and sequential relationships in malware behavior, allowing the model to generalize more effectively to unseen malware variants. The flexibility of our approach enhances its ability to detect novel malware, even those that exhibit behavior outside of the training data distribution.

**Reviewer#6 Comment 3:** Ensure consistent use of terms like "graph convolutional networks (GCN)" and "directed graph convolutional networks (DGCN)" across sections to avoid confusion.

**Response:**

Thank you very much for your valuable feedback and for pointing out the importance of consistent terminology usage within our manuscript. We acknowledge that maintaining consistency in the terms used is crucial for clarity and to avoid any potential confusion for the readers.

In response to your comment, we have carefully reviewed the entire manuscript and ensured that the terms "graph convolutional networks (GCN)" and "directed graph convolutional networks (DGCN)" are used consistently across all sections. Specifically, we have:

(1) Conducted a thorough search for all instances where these terms appear.

Verified that the full form is introduced before or at the first mention of the acronym in the text.

(2) Corrected any variations in the usage of these terms to ensure uniformity.
(3) Checked for any potential ambiguity and clarified the context where necessary.

We believe these changes will enhance the readability and precision of our paper. We appreciate your attention to this detail and hope that our revisions meet with your approval.

**Reviewer#6 Comment 4:** Some references, such as those for malware statistics, could be better integrated into the discussion to highlight their relevance to the problem statement.

**Response:**

We greatly appreciate your thoughtful feedback, especially regarding the integration of malware statistics and the importance of clearly articulating the relevance of these figures to the research problem. In response, we have made several changes to the introduction to better align these statistics with the challenges in malware detection.

(1) Integration of Malware Statistics: As per your suggestion, we have enhanced the connection between the provided malware statistics (such as the increase in ransomware incidents and overall malware activity) and the growing complexity of malware threats. We now explicitly highlight how these statistics underscore the escalating volume and sophistication of malware, directly motivating the need for more advanced malware detection methods. For example, we now emphasize that the 13% year-on-year increase in ransomware incidents (according to the Verizon Business 2022 Data Breach Investigations Report) highlights an urgent need for effective and adaptive malware detection techniques.
(2) Clearer Articulation of the Research Problem: The discussion now more clearly emphasizes how the growing diversity and sophistication of malware types make traditional detection methods insufficient. We connect these challenges to the limitations of static and traditional machine learning methods, making a stronger case for the need for more sophisticated, dynamic techniques—such as our novel approach using directed graph representations of API call sequences.

We believe these revisions will provide a more coherent narrative that effectively ties together the problem context, the limitations of existing approaches, and the motivation for our proposed solution. Thank you again for your valuable input, and we look forward to your further feedback.

**Reviewer#6 Comment 5:** Ensure all abbreviations like FSADGCN, CNN, and API are defined explicitly when first introduced. For example, "API sequence instructions" can be expanded to "Application Programming Interface (API) sequence instructions."

**Response:**

Thank you for your meticulous review and the valuable suggestion to ensure that all abbreviations are explicitly defined upon their first introduction in the manuscript. We agree that this practice enhances the clarity and accessibility of our paper, especially for readers who may not be familiar with the specific terminologies used.

In response to your comment, we have made the following adjustments:

(1) Explicit Definition of Abbreviations: We have reviewed the entire document and identified all instances where abbreviations such as FSADGCN, CNN, and API are used. For each abbreviation, we have provided a full expansion at its first appearance in the text. For example, "API sequence instructions" has been revised to "Application Programming Interface (API) sequence instructions."
(2) Consistency Check: In addition to defining abbreviations, we have ensured that they are used consistently throughout the manuscript. This means that once an abbreviation is introduced, it is used in subsequent mentions, unless the context requires the full term for clarity.
(3) Verification: To prevent any oversight, we have conducted a final verification to confirm that no abbreviations are left undefined and that all expansions are accurate and appropriate.

We believe these changes will significantly improve the clarity and comprehensibility of our work. We appreciate your attention to detail and thank you for your constructive feedback.

**Reviewer#6 Comment 6:** Replace the placeholder text "Fig 1 venenatis sed ipsum varius..." with an appropriate description or remove unrelated filler text. Ensure figure references are meaningful and properly aligned with the content.

**Response:**

Thank you for your valuable feedback regarding the placeholder text in Figure 1 and the importance of meaningful figure references. We appreciate your attention to detail, which has helped us improve the clarity and coherence of our manuscript. In response to your comment, we have made the following revisions:

(1) Replacement of Placeholder Text:

We have replaced the placeholder text "Fig 1 venenatis sed ipsum varius..." with a clear and detailed description of Figure 1. The new caption now accurately reflects the content and purpose of the figure, providing readers with a comprehensive understanding of the illustrated process.

(2) Enhanced Figure Description:

For Figure 1, the updated caption reads: "Workflow of the proposed malware classification method based on FSGCN. This figure illustrates the process from handling imbalanced datasets using the SMOTE algorithm, constructing directed graphs for API sequences, extracting multi-dimensional attribute features, computing first-order and second-order adjacency matrices, generating feature embeddings with GCN, converting these embeddings into grayscale images, and finally classifying the images using CNN."

We believe these changes will significantly enhance the clarity and impact of our paper. We appreciate your constructive comments and hope that the revised manuscript meets with your approval.

**Reviewer#6 Comment 7:** Rephrase sentences to improve readability, such as changing "The extracted node feature attributes are then subjected to..." to "Next, the extracted node feature attributes undergo..."

**Response:**

Thank you for your valuable feedback. We have carefully considered your suggestion to improve sentence readability, particularly the one regarding "The extracted node feature attributes are then subjected to...". We agree that using more direct and concise language improves clarity and flow. As recommended, we have rephrased this sentence to "Next, the extracted node feature attributes undergo...," which simplifies the construction and enhances readability without altering the meaning.

Additionally, we have applied similar revisions throughout the paper to ensure a consistent and formal tone, enhancing overall readability and making the text more accessible to readers. We appreciate your insights and believe these adjustments improve the quality of the manuscript.

**Reviewer#6 Comment 8:** Review and adjust the formatting of equations to ensure clarity and consistency, such as spacing and alignment in equations (e.g., Equations 1-11). Use inline or displayed math consistently to improve readability.

**Response:**

Thank you for your thoughtful review. We appreciate your recommendation to review and adjust the formatting of the equations to improve clarity and consistency. We understand the importance of clear and well-aligned mathematical expressions, and we have made the necessary revisions to ensure that all equations (e.g., Equations 1-11) are properly spaced and aligned.

As per your suggestion, we have ensured that:

(1) Spacing: We have adjusted the spacing within and around the equations to ensure readability and consistency. For instance, we have ensured consistent use of spacing between variables, operators, and functions.
(2) Alignment: Equations that span multiple lines have been aligned correctly to enhance visual clarity, making them easier to follow.
(3) Math Formatting: We have reviewed our use of inline and displayed math expressions and ensured they are used consistently throughout the paper. Displayed equations are now reserved for more complex expressions, while inline math is used for simpler notations to improve readability and flow.

Since we used the LaTeX template provided by the journal, we have made sure that the formatting adheres to the journal's guidelines while implementing the adjustments you suggested.

Thank you again for your helpful feedback, which we believe enhances the overall presentation and readability of our manuscript.

**Reviewer#7 Comment 1:** Clearly mention the improvements in the abstract section.

**Response:**

Thank you for your valuable feedback. We appreciate your suggestion to clearly highlight the improvements in the abstract section. In response to your comment, we have revised the abstract to explicitly emphasize the key advancements and contributions of our work. The updated abstract now clearly outlines the innovations in our approach, particularly focusing on how our method addresses the limitations of existing malware classification techniques by incorporating directed API call relationships, utilizing GCN for feature extraction, and applying the SMOTE algorithm for handling imbalanced datasets.

By highlighting these improvements, we aim to provide readers with a concise and clear understanding of the novel contributions of our research right from the abstract.

Thank you again for your insightful recommendation, which has helped us refine the presentation of our work.

**Reviewer#7 Comment 2:** The image quality of Figure 4 needs improvement as the text is unclear, and the color scheme for the bars could be enhanced for better visual distinction.

**Response:**

Thank you for your detailed feedback on the image quality of Figure 4 and the suggestions for improving its visual presentation. We fully agree that the clarity of the text and the color scheme are critical for effective communication of our findings.

In response to your comments, we have taken the following actions:

(1) Image Quality Enhancement: We have reprocessed Figure 4 using higher resolution graphics to ensure that all textual elements are clear and legible. This should improve the overall readability of the figure.
(2) Color Scheme Optimization: We have revised the color scheme for the bars in Figure 4 to provide better visual distinction between different categories. We have chosen a set of colors that not only enhance contrast but also adhere to color accessibility guidelines, ensuring that the figure is accessible to all readers。

We believe these changes will significantly improve the interpretability and visual appeal of Figure 4, thereby enhancing the overall quality of the manuscript. Your constructive criticism has been invaluable in helping us refine our work.

**Reviewer#7 Comment 3:** Presenting the dataset description in a tabular format in section could more effectively showcase the dataset statistics.

**Response:**

Thank you for your valuable feedback regarding the presentation of the dataset description. We agree that showcasing the dataset statistics in a tabular format within the relevant section can enhance the clarity and accessibility of the information for readers.

In response to your suggestion, we have revised the manuscript to include a detailed table (Table 2) that encapsulates the overall situation and key characteristics of the datasets. This adjustment aims to provide a more structured overview, allowing for a more effective comparison and understanding of the data used in our study.

We believe this change significantly improves the presentation of our dataset and aids in better communicating our research to the academic community. Your insight has been instrumental in refining our work.

We trust that these responses and the associated revisions thoroughly address your concerns and enhance the manuscript's quality. We are grateful for the opportunity to refine our work and eagerly await any further feedback.

Best regards,

[Cuihua Ma,Zhenwan Li,Haixia Long,Anas Bilal,Xiaowen Liu]
